# Neuromodulation for recovery of trunk and sitting functions following spinal cord injury: a comprehensive review of the literature

**DOI:** 10.1186/s42234-023-00113-6

**Published:** 2023-05-29

**Authors:** Niraj Singh Tharu, Arnold Yu Lok Wong, Yong-Ping Zheng

**Affiliations:** 1 Department of Biomedical Engineering, The Hong Kong Polytechnic University, Hong Kong SAR, China; 2 Department of Rehabilitation Sciences, The Hong Kong Polytechnic University, Hong Kong SAR, China; 3 Research Institute for Smart Ageing, The Hong Kong Polytechnic University, Hong Kong SAR, China

**Keywords:** Neuromodulation, Trunk stability, Sitting balance, Rehabilitation, Spinal cord injury

## Abstract

Trunk stability is crucial for people with trunk paralysis resulting from spinal cord injuries (SCI), as it plays a significant role in performing daily life activities and preventing from fall-related accidents. Traditional therapy used assistive methods or seating modifications to provide passive assistance while restricting their daily functionality. The recent emergence of neuromodulation techniques has been reported as an alternative therapy that could improve trunk and sitting functions following SCI. The aim of this review was to provide a broad perspective on the existing studies using neuromodulation techniques and identify their potentials in terms of trunk recovery for people with SCI. Five databases were searched (PubMed, Embase, Science Direct, Medline-Ovid, and Web of Science) from inception to December 31, 2022 to identify relevant studies. A total of 21 studies, involving 117 participants with SCI, were included in this review. According to these studies, neuromodulation significantly improved the reaching ability, restored trunk stability and seated posture, increased sitting balance, as well as elevated activity of trunk and back muscles, which were considered early predictors of trunk recovery after SCI. However, there is limited evidence regarding neuromodulation techniques on the improvement of trunk and sitting functions. Therefore, future large-scale randomized controlled trials are warranted to validate these preliminary findings.

## Background

Spinal cord injury (SCI) often causes paralysis of the extremities and trunk muscles, which impairs functional control of the trunk and sitting balance, resulting in trunk instability (Bao et al. 2020). Trunk instability is a major concern for people with SCI (Milosevic et al. 2015a). Particularly, lesions in the cervical to the thoracic region can paralyze trunk muscles, leading to a partial or complete loss of trunk stability (Friederich et al. 2020). Such impairments adversely affect an individual’s ability to carry out everyday activities (Patel et al. 2017), including bed movements, unsupported sitting, and self-care duties (Triolo et al. 2009). Additionally, trunk instability could lead to various secondary health issues such as pressure injuries (Tharu et al. 2022a), spinal deformities (Friederich et al. 2020), and pulmonary dysfunction (Patel et al. 2017). Approximately 70–80% of SCI survivors have decreased or no trunk control (Rahimi et al. 2020), and are wheelchair-bound (Tharu et al. 2022b). Paralysis of trunk muscles is one of the most significant factors affecting sitting balance in individuals with SCI (Bergmann et al. 2019; Friederich et al. 2020), which limit their capacity to perform transfers, propel their manual wheelchairs, and reach for objects (Triolo et al. 2009). People with SCI are also at a higher risk of falls even during stationary sitting, which may lead to fall-related pain, bone fractures, and other injuries (Rath et al. 2018). As such, the quality of life in people with SCI depends on their dynamic sitting balance and the ability to sit unsupported (Bergmann et al. 2019), which becomes more important as the post-injury time increases (Friederich et al. 2020). Therefore, trunk recovery is an essential factor for those with sitting difficulties (Tharu et al. 2022b). Unfortunately, only a few studies have investigated the sitting balance or trunk stability in people with SCI (Bergmann et al. 2019).

Belts, straps, or customized seating adaptations are conventionally used to maintain sitting stability of people with SCI. They were often used to stabilize the trunk to avoid falls, and to assist the effective usage of upper limbs (Triolo et al. 2013a). However, such techniques reduced the trunk’s dynamic mobility and to reach for objects (Friederich et al. 2021). Neuromodulation is an emerging viable treatment option for paralysis following SCI (Seáñez and Capogrosso, 2021; Rahman et al. 2022). Various forms of neuromodulation have been used to treat SCI, such as functional electrical stimulation (FES) (Wilkenfeld et al. 2006b), electrical stimulation (ES) (Momeni et al. 2016), functional neuromuscular stimulation (FNS) (Friederich et al. 2022), transcutaneous electrical spinal cord stimulation (TSCS) (Tharu et al. 2022b), and epidural spinal electrical stimulation (EES) (Gill et al. 2020). FES, ES and FNS can be delivered both invasively and non-invasively. FES showed the potential to recover certain dynamic trunk functions (Friederich et al. 2020), while ES and FNS improved upright sitting posture (Triolo et al. 2013b; Friederich et al. 2021). Recent research also reported that using TSCS and EES might improve post-SCI motor recovery and are safe and well tolerated (Lin et al. 2022). Studies showed that some people with SCI regained upper and lower limb motor control following neuromodulation (FES, TSCS, or EES) treatment (Kapadia et al. 2011; Alam et al. 2020; Greiner et al. 2021; Gorgey and Gouda 2022; McGeady et al. 2022). Motor functions modulated by TSCS and EES could restore trunk and sitting impairments (Laskin et al. 2022). However, only a limited number of studies have examined the possibility of trunk recovery after SCI (Rath et al. 2018; Chiou and Strutton 2020; Tharu et al. 2022b), but due to their small sample size the effects remain uncertain and even fewer have examined the effects of TSCS (Rath et al. 2018; Tharu et al. 2022b) or EES (Triolo et al. 2009) for trunk and sitting improvement.

FES and ES are commonly used for muscle strengthening by directly stimulating the target muscle (Bergmann et al. 2019; Kouwijzer et al. 2022), whereas FNS is applied over the nerve supply of the affected muscles to induce muscle contraction (Friederich et al. 2020). TSCS is a non-invasive transcutaneous approach applying to the skin overlying the spinal cord (Tharu et al. 2022b), while EES uses multichannel electrodes placed invasively on the dorsal epidural surface of the spinal cord (Gill et al. 2020). FES and ES use similar stimulation techniques for similar purposes. A slight difference is that ES activate muscles through stimulation of intact peripheral motor nerves, while FES is the use of neuromuscular electrical stimulation to promote functional activities (Johnston 2016). TSCS and EES techniques focus on sensorimotor functions, including the activation of the paralysed muscle caused by SCI (Rath et al. 2018; Rowald et al. 2022). They can achieve similar treatment effects, with EES being invasive and TSCS non-invasive (Rahman et al. 2022; Gill et al. 2020). We noted that different terms have historically been used by different research groups for similar techniques, and it may be necessary to standardize the terminology according to whether the electrodes are implanted and what the electrical siginal is used to simulate, spinal cord, peripheral nerve, or muscle.

The aim of this review was to summarize research investigating the effects of the five types of neuromodulation (FES, ES, FNS, TSCS and EES) in improving trunk and sitting functions in people with SCI, and discuss the limitations and future research direction in this intriguing field. This review may help readers better understand the effects of neuromodulation on trunk and sitting functions, and develop alternative rehabilitation strategies, instead of focusing on peripheral limb functions (Seáñez and Capogrosso, 2021).

## Methods

### Search strategy

Relevant titles and abstracts were searched from PubMed, Embase, Science Direct, Medline (Ovid), and Web of Science. Articles were searched from the inception until December 31, 2022. After reading the abstract and relevant articles were retrieved for full-text screening. The identified articles were double checked before the data extraction. Backward searches of the reference lists of the included articles were conducted. The database search results were transferred to EndNote version 20.2.1 to remove duplicates and for further processing.

The major keywords and medical subject headings used for the search were: neuromodulation, invasive stimulation, non-invasive stimulation, implanted stimulation, transcutaneous electrical spinal cord stimulation, epidural spinal cord stimulation, functional electrical stimulation, electrical stimulation, neuromuscular stimulation, trunk control, sitting control, trunk stability, sitting stability, trunk function, sitting function, trunk balance, sitting balance, rehabilitation, paralysis, paraplegia, quadriplegia, tetraplegia, spinal cord injury, and spinal cord disease.

### Screening procedure

The titles and abstracts of the identified citations were independently screened based on the selection criteria. The results were counterchecked after screening and potential full-text articles were extracted and screened using the same procedure.

### Inclusion and exclusion criteria

Studies were included if they involved: (1) people with SCI; (2) at least one type of neuromodulation; and (3) primary outcomes related to trunk and sitting functions. The exclusion criteria were: (1) animal research; (2) able-bodied individuals; (3) reliability or validity studies; (3) investigating exoskeletons or robotics; (5) involving only therapeutic exercise; (6) investigating trunk muscle atrophy, tissue health, gluteal pressure, or muscle synergies; or (7) assessing trunk stability during standing, walking, or locomotion, or upper extremity function.

### Data extraction and synthesis

The data extraction was performed and randomly 50% of the articles were selected to check for accuracy and consistency of the extracted data. Any discrepancies were discussed and corrected. Data extraction was repeated for articles that had more than 5% discrepancy during the quality assurance check. The extracted data included: authors, year of publication, study design, neuromodulation type, sample size, participants’ characteristics (SCI type, level of SCI, American Spinal Injury Association Impairment Scale (AIS) grade), stimulation region and parameters, specific outcomes, and adverse effects. The extracted data were tabulated and/or put in figures for all five types of neuromodulation.

## Results

### Neuromodulation characteristics

**Fig. 1:**
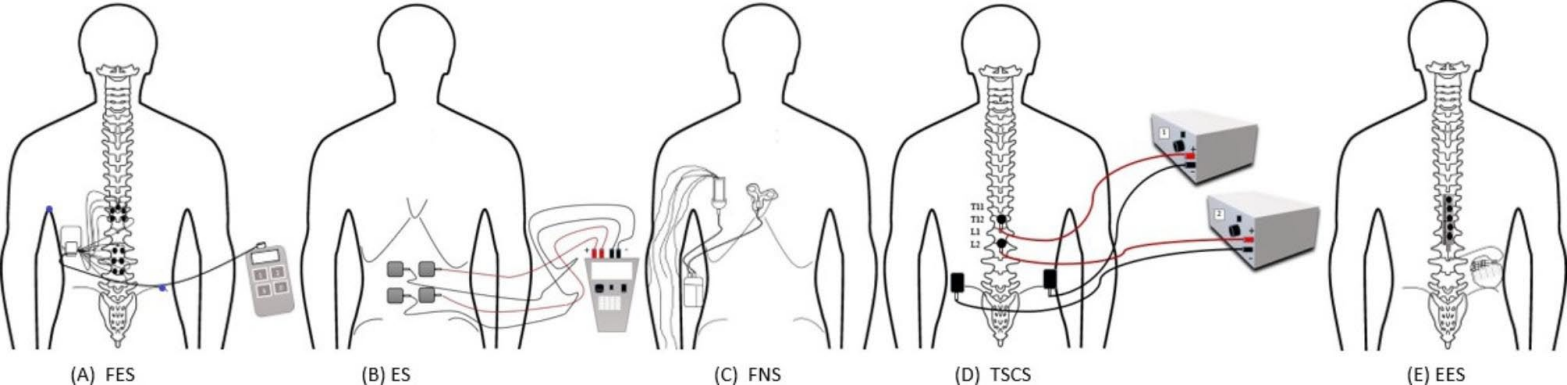
Five forms of neuromodulation therapies (**A**) FES, (**B**) ES, (**C**) FNS, (**D**) TSCS, and (**E**) EES, described in this review. Electrodes placement: FES implanted in thoraco-lumbar region, ES transcutaneously over abdominal muscles, FNS implanted in thoracic region, TSCS transcutaneously over spinal cord in thoraco-lumbar region, and EES implanted in dorsal epidural surface in lumbosacral spinal cord enlargement. Abbreviation: FES = functional electrical stimulation; ES = electrical stimulation; FNS = functional neuromuscular stimulation; TSCS = transcutaneous electrical spinal cord stimulation; EES = epidural spinal electrical stimulation

Various neuromodulation methods identified in this review are shown in Fig. 1. FES was administered transcutaneously over the targeted muscle fibers or invasively implanted at the neural circuits to trigger detectable muscle contraction (Rahman et al. 2022). When compared to ES and FNS, FES is preferable because it not only increases muscular activation but also improves muscle recruitment (Bergmann et al. 2019). In addition, it may induce muscular contraction by delivering short electric pulses between adjacent electrodes (Milosevic et al. 2015b). ES was delivered through a portable stimulator with self-adhesive electrodes (Kouwijzer et al. 2022), as well as through an implanted-receiver telemeter placed at the spinal roots of the innervated muscles. Further, electrode leads were inserted subcutaneously and attached to a custom multichannel pulse generator, which was operated by a powered external wearable microprocessor-based controller (Triolo et al. 2009, 2013a, b). For FNS, electrodes placed on the surface of the skin or surgically placed at the motor points of the targeted muscles to induce muscle contraction (Audu et al. 2015; Friederich et al. 2020). Additionally, a neuroprosthesis made up of a stimulator-telemeter was implanted using epimysial or intramuscular electrodes (Friederich et al. 2021, 2022). The stimulation method for FNS differs from FES and ES in that it includes a proportional-integral-derivative controller (Friederich et al. 2021) coupled to intramuscular, epimysial, or nerve cuff electrodes that are invasively implanted to stimulate the nerves that supply the muscle groups (Friederich et al. 2022). Furthermore, FES, ES and FNS followed similar stimulation process for the non-invasive method where the target was to elicit muscle contraction through placement of surface electrodes whereas, their procedures differed during the invasive technique where ES used additional implanted-receiver telemeter and FNS utilized proportional-integral-derivative controller (Friederich et al. 2021) to improve their outcomes in comparison to FES. However, FES was found to have better functional results than ES and FNS. TSCS is a non-invasive technique that targets the spinal cord using electrodes that are attached superficially to the skin over the spine (Rahman et al. 2022). EES is an invasive technique in which electrical stimulation is administered to the dorsal epidural surface of the spinal cord using a stimulation electrode array that is implanted to the lumbosacral spinal cord enlargement to elicit evoked motor potentials (Gill et al. 2020). It employs multielectrode paddle leads originally developed to target the spinal cord’s dorsal column (Rowald et al. 2022).

### Articles retrieved

**Fig. 2:**
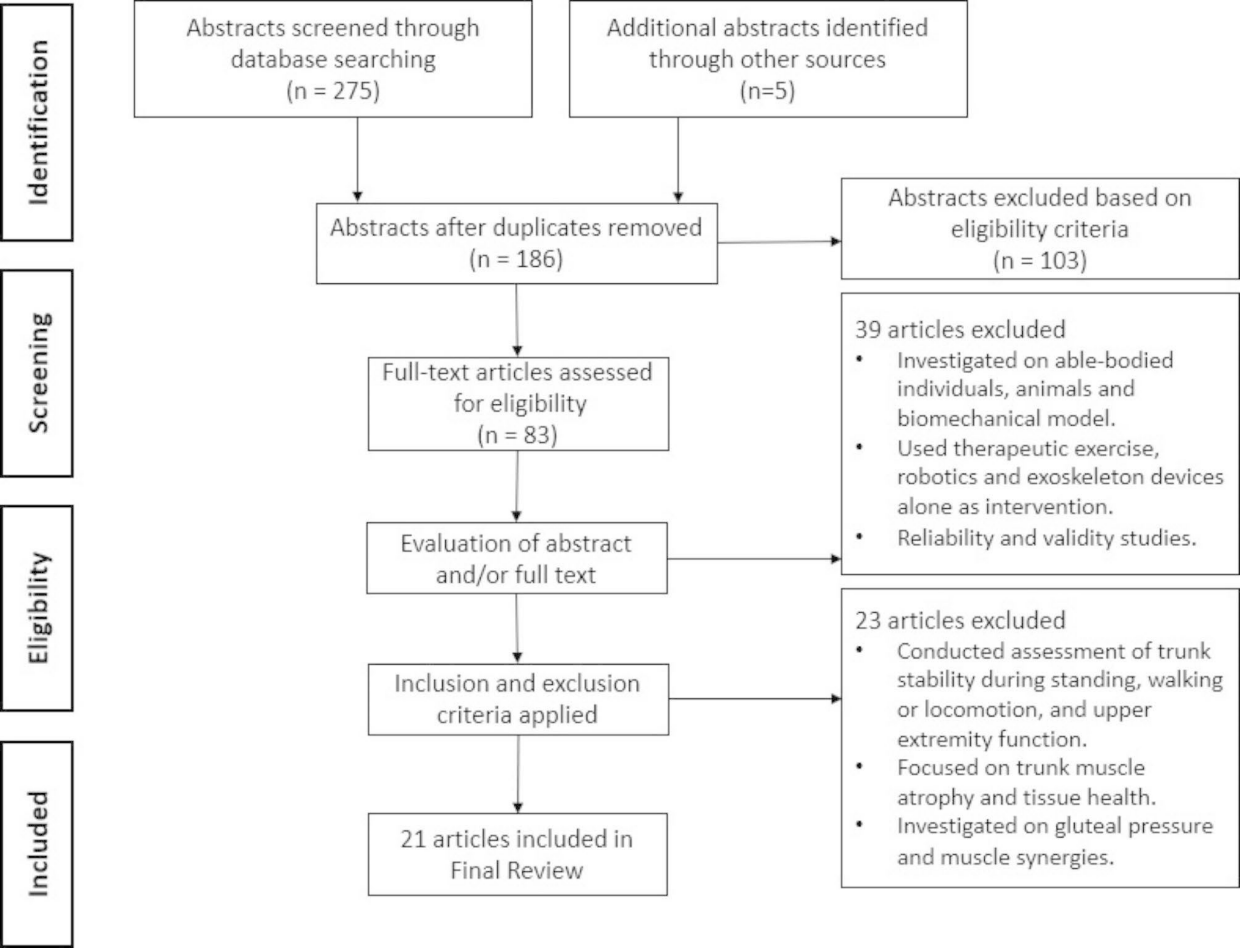
Flow diagram of the article selection process

Databases were searched and 94 duplicates were removed using EndNote. 275 abstracts were screened followed by an additional five abstracts were identified from the reference lists of the included articles. One hundred and three abstracts were excluded based on the eligibility criteria. Furthermore, 83 full-text articles were assessed for eligibility and 62 full-text articles were excluded because they investigated able-bodied individuals, animals and biomechanical models, their primary assessment focused on upper extremity, standing and locomotion function, or examined gluteal pressure, tissue health and muscle synergies. Twenty-one articles were included (FES = 5, ES = 5, FNS = 5, TSCS = 3, and EES = 3). The study selection process is summarized in Fig. 2.

### Publication trends of neuromodulation studies

**Fig. 3:**
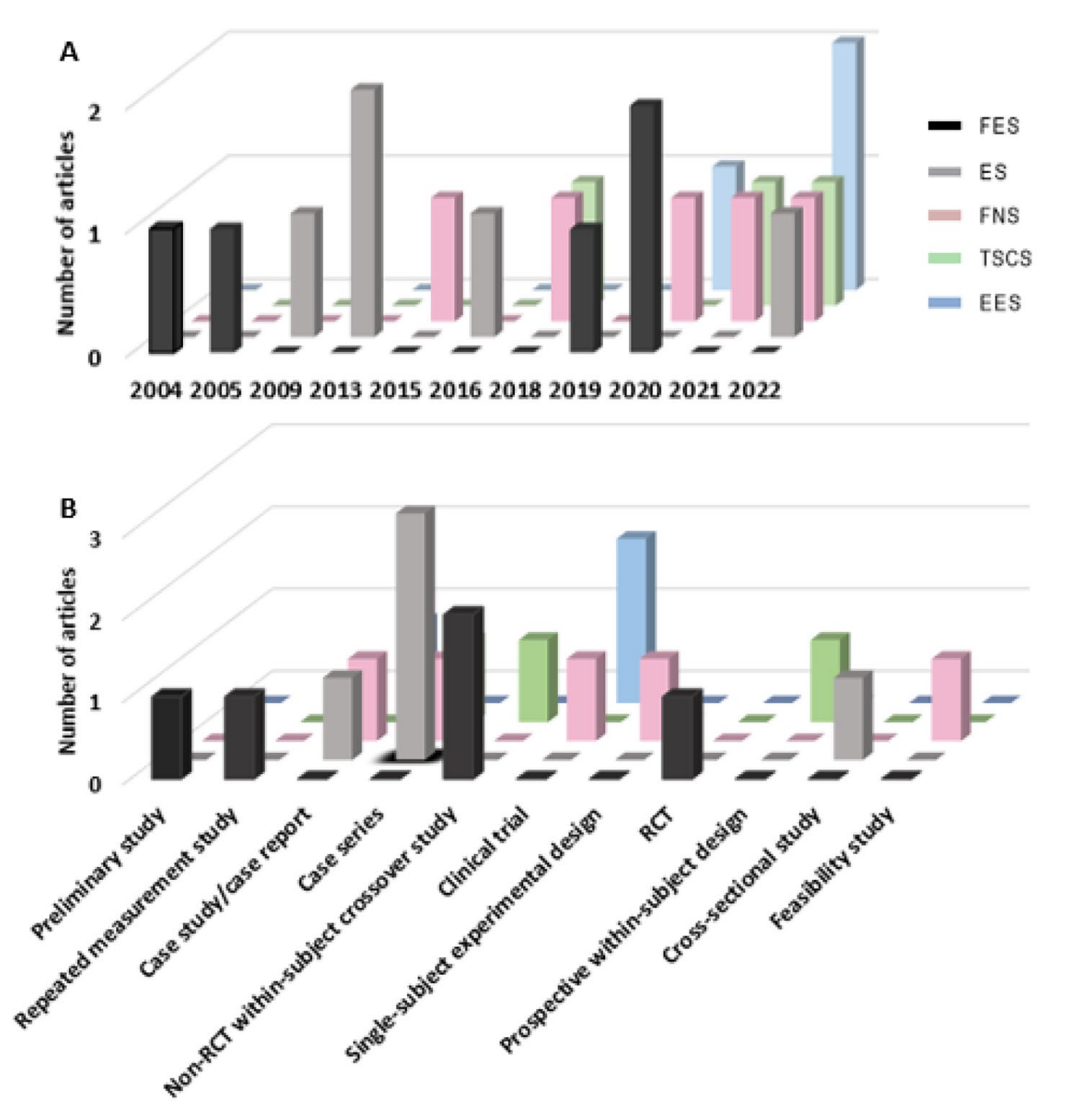
(**A**) Publication trends for FES (n = 5), ES (n = 5), FNS (n = 5), TSCS (n = 3), and EES (n = 3) by year; and (**B**) reported study designs of each neuromodulation technique with number of articles published. Abbreviation: FES = functional electrical stimulation; ES = electrical stimulation; FNS = functional neuromuscular stimulation; TSCS = transcutaneous electrical spinal cord stimulation; EES = epidural spinal electrical stimulation; RCT = randomized controlled trial

As shown in Fig. 3A, the earliest article was published in 2004 to investigate the effect of implanted FES on lumbar trunk extensor in sitting among people with SCI (Kukke and Triolo 2004). In 2005, transcutaneous FES was used to investigate trunk musculature during wheelchair propulsion (Yang 2005). During 2019 and 2020, two additional non-randomised controlled cross-over studies and one randomized controlled trial (RCT) separately investigated the effects of transcutaneous FES on trunk muscle tone, sitting balance, and transfer abilities (including attaining independence in activities of daily living) in people with SCI (Bergmann et al. 2019, 2020; Rahimi et al. 2020). Two case series and one case study published between 2009 and 2013 investigated the effects of implanted ES on stabilizing the paralyzed trunk, seated function, and reaching, respectively (Triolo et al. 2009, 2013a, b). Similarly, two case series and one cross-sectional study published in 2016 (Momeni et al. 2016; Tharu et al. 2022b) and 2022 (Kouwijzer et al. 2022) evaluated the effects of transcutaneous ES on trunk stability and trunk muscle activation. Additionally, one single-subject experimental study, one case study, one feasibility study, a non-RCT clinical trial, and a case series published between 2015 and 2022 investigated the effect of implanted FNS on sitting stability, wheelchair propulsion, upright sitting recovery, force production capabilities of paralyzed trunk muscles, and functional reaching task performance (Audu et al. 2015; Armstrong et al. 2018; Friederich et al. 2020, 2021, 2022). One within-subject crossover study, prospective within-subject study, and case series, published in 2018 (Rath et al. 2018), 2021 (Keller et al. 2021), and 2022 (Tharu et al. 2022b), respectively, examined the effectiveness of TSCS in improving trunk stability, upright trunk posture, trunk control, and sitting functions. Further, one non-RCT clinical trial and a case report published between 2021 and 2022 investigated the effect of EES on trunk stability during seated reaching performance, trunk control, and trunk functions after chronic SCI (Gill et al. 2020; Gorgey and Gouda 2022; Rowald et al. 2022).

### Study design

There was difficulty in identifying the study design due to the insufficient details reported. Figure 3B, showed that case series (5 of 21 articles) was the most common type of study design of the included articles. Furthermore, non-randomized within-subject crossover study (3 of 21 articles), non-RCT clinical trial (3 of 21 articles), and case study (2 of 21 articles) were other commonly used study designs. Other included study designs included: preliminary study, repeated measurement study, non-RCT feasibility study, case report, and cross-sectional study. ES was commonly investigated by case series, whereas non-randomized within-subject crossover study design was used in FES-related studies. Single-subject experimental design was frequently used for FNS investigation. Moreover, TSCS and EES were studied using several study designs (Table 1). Of the reviewed articles, there was only one randomized controlled trial that investigated the effects of weight bearing exercises with or without FES on the selected trunk muscle contraction ability to transfer, and attain independence in activities of daily living among wheelchair-bound people with SCI.

**Table 1:** Characteristics of 21 included studies

Study and Year	Design	Subjects (n)	Age (yr) mean ± SD	Level of injury and AIS classification	Post-SCI (yr)	Neuromodulation type	Stimulation setting	Stimulation region	Assessment	Outcomes
(Kukke and Triolo, 2004)	Preliminary study	n = 4; M = 3 F = 1	35.2 ± 9.2	C7 - T8; AIS A − 2 AIS B − 2	6.7 ± 6.8	Implanted functional electrical stimulation	Pulse duration 0-200 µs and increased to greatest amount until trunk extension was observed.	Intramuscular electrodes implanted between L1–L2 or T12–L1 spinal segments.	Motion capture system, bimanual reaching maneuvers, sagittal reaching length.	Improvement in seated posture and increased bimanual reaching distance.
(Yang 2005) (2005)	Repeated measurement study	n = 12; M = 10 F = 2	41.6 ± 9.1	C6 - T10; AIS A − 8 AIS B − 3 AIS C-1	17.5 ± 8.5	Functional electrical stimulation	Biphasic stimulation, frequency 30 Hz, pulse width 300 µs, amplitude 80 mA.	Abdominal and back muscles stimulated.	Electromyography, wheel chair propulsion, trunk flexion, maximum voluntary contraction.	Abdominal and back muscles were highly activated, trunk stability increased.
(Triolo et al. 2009) (2009)	Case study	n = 1; M	40	C4 AIS - A	20	Implanted electrical stimulation	Biphasic stimulation, stimulus amplitude (0.1–20 mA), pulse duration (0-255 µs), frequency 14 Hz.	L1 and T12 spinal roots.	Forward bimanual reaching distance, seated stability, rolling in bed without assistance.	Improved forward reach, restoration of upright sitting and bed turning was also improved.
(Triolo et al. 2013a)	Case series	n = 6; M = 4 F = 2	46 ± 10.8	C6 - T10; AIS A − 3 AIS B − 2 AIS C-1	8.6 ± 2.8	Implanted electrical stimulation	Biphasic stimulation, intensity 20 mA, frequency 20 Hz, pulse width 0-250 µs.	L1 - L2 spinal nerves.	Maximum forward trunk lean, pushrim kinematics (peak shoulder moment and propulsion)	Increase in forward reach by 19–26% stabilizing the trunk.
(Triolo et al. 2013b)	Case series	n = 8; M = 6 F = 2	46 ± 9.9	C5 - T10; AIS A − 3 AIS B − 3 AIS C-2	11.5 ± 6.9	Implanted electrical stimulation	Biphasic stimulation, stimulus amplitude (2–20 mA), frequency 20 Hz, pulse width 0-250 µs.	L1 - L2 spinal nerves.	Trunk extension strength, seated stability, bimanual reaching.	Increase in trunk extension and forward reach distance with improved sitting posture.
(Audu et al. 2015)	Case series	n = 5; M = 3 F = 2	53.4 ± 7.7	C7 - T10; AIS − 2 AIS B − 2 AIS C − 1	10 ± 4.3	Implanted functional neuromuscular stimulation	Frequency 20 Hz, 50 ms, interpulse interval.	Hip and back extensor muscles stimulated.	Seated balance under external perturbations (forward flexion), trunk tilt, erect posture.	Improved forward trunk tilt flexion and erect posture.
(Momeni et al. 2016)	Case series	n = 3	21.0 ± 1.0	AIS - B	10 ± 3.5	Electrical stimulation	Biphasic pulses of 300 µs at 35 Hz, Intensity increased till visible muscle contraction.	Rectus femoris, biceps femoris, gastrocnemius, and tibialis anterior.	Surface electromyography, 10-meter walk test.	Trunk muscle activation and improved trunk stability.
(Rath et al. 2018)	Non-RCT within-subject crossover study	n = 8; M = 7 F = 1	29.9 ± 7.7	C4 - T9; AIS A − 6 AIS C − 2	7.5 ± 3.3	Transcutaneous electrical spinal cord stimulation	Monophasic rectangular 1ms pulses, frequency 30 Hz at T11, and 15 Hz at L1, carrier frequency 10 kHz, intensity 10 to 150mA	Between T11 and T12; and between L1 and L2 hereafter referred to as T11 and L1.	Electromyography of the trunk muscles, three-dimensional kinematics, and force plate data were acquired.	Elevated activity of the trunk muscles contributing to improved trunk control, and increased multi-directional seated stability.
(Armstrong et al. 2018)	Clinical trial	n = 4; M = 2 F = 2	48.7 ± 8.0	C7 - T4; AIS A -2 AIS B − 2	13.0 ± 6.2	Functional neuromuscular stimulation	Pulse amplitudes (0 to 20 mA), pulse durations (0 to 250 µsec), frequency (0 to 20 Hz).	Inserted at T12-L2 spinal nerves to activate the paraspinal muscles.	Turning events, superior-inferior angular velocity and displacement of trunk and pelvis measured.	Activation of the paraspinal and hip muscles, recovery of upright sitting, restoring a stable and erect posture.
(Bergmann et al. 2019)	Non-RCT Crossover study	n = 5; M	39.2 ± 7.1	C5-C6; AIS B − 4 AIS C − 1	10.8 ± 6.0	Functional electrical stimulation	Frequency 3–18 Hz, pulse width 275 µs, intensity increased till strong visible muscle contraction.	Erector spinae and rectus abdominis muscles.	Muscle oscillation frequency, characterizing muscle tone, limits of stability, and characterizing sitting balance were measured.	Increased trunk muscle tone and improved dynamic sitting balance during flexion movement.
(Friederich et al. 2020)	Single-subject experimental design	n = 4; M = 2 F = 2	50.7 ± 8.3	C5-T4; AIS A − 1 AIS B − 2 AIS C − 1	11.2 ± 6.9	Functional neuromuscular stimulation	Pulse width (0-250 µs), stimulus amplitude (2–20 mA), frequency 20 Hz.	Set of trunk muscles stimulated.	Electromyography, isometric muscle contraction.	Stimulated muscles were activated with increase in muscle force.
(Bergmann et al. 2020)	Non-RCT crossover study	n = 5; M	39.0 ± 7.0	C5 - C6; AIS B – 4 AIS C − 1	10.8 ± 6.0	Functional electrical stimulation	Frequency 8–18 Hz, pulse width 275 µs, intensity increased till strong visible muscle contraction.	Placed on thoraco-lumbar area of the erector spinae and rectus abdominis muscles bilaterally.	EMG (maximum voluntary isometric contraction), manual muscle test hand-held dynamometer.	Improved trunk muscle force generation and muscle fatigue reduced.
(Gill et al. 2020)	Clinical trial	n = 2; M	31.5 ± 7.8	T3 & T6 AIS - A	4.5 ± 2.1	Epidural spinal electrical stimulation	Frequency 20–25 Hz, pulse width 200–400 µsc, stimulation intensity 3.8–5.0 V.	T11 - L1 vertebral region.	Reaching performance, modified functional reach test.	Improved reaching performance and seated position, increase in reaching distance.
(Rahimi et al. 2020)	Randomised controlled trial	n = 16; M = 13 F = 3	37.0 ± 5.7	T5 - T12; AIS - A	13.0 ± 5.7	Functional electrical stimulation	Rectangular pulses, pulse width 400 µs, frequency 40 Hz, amplitude 20 to 200 mA increased to a visible contraction.	Quadriceps and gastrocnemius muscles.	Spinal Cord Independence Measure-III, quadruped unilateral reaching.	Improved ability to perform transfers, increased unilateral reaching.
(Keller et al. 2021)	Prospective within-subject design	n = 8; M = 5 F = 3	8.4 ± 3.9	Cervical-thoracic	NA	Transcutaneous electrical spinal cord stimulation	Frequency 30 Hz, intensity 134–140 mA.	T11 and L1 spinal levels.	Electromyography, trunk kinematics, center of pressure displacement, segmental assessment of trunk control.	Increased trunk extension, enabled upright sitting posture.
(Friederich et al. 2021)	Case study	n = 1; F	48	C 7 AIS - B	22	Functional neuromuscular stimulation	Frequency 20 Hz, pulse 250 µs and amplitude set at 20mA.	Applied to nerves innervating the lumbar erector spinae, quadratus lumborum, adductor magnus, gluteus maximus, gluteus medius, and hamstring semimembranosus.	Trunk tilt, functional tasks in sitting, motion capture system.	Increased trunk movement and improved erect upright sitting posture.
(Tharu et al. 2022b)	Case series	n = 5; M = 2 F = 3	42 ± 13.7	C4 - C7; AIS - A	9.3 ± 7.4	Transcutaneous electrical spinal cord stimulation	Biphasic stimulation, Frequency 20–30 Hz, pulse width 0.1-1.0 ms, intensity 90–115 mA.	T11-T12 and L1-L2 spinal levels.	Functional reach test, trunk control test and function in sitting test, electromyography, motion capture system.	Improved trunk and sitting functions with increased static and dynamic balance.
(Kouwijzer et al. 2022)	Cross-sectional study	n = 11; M = 10 F = 1	41.6 ± 10.1	C4 - C7; Complete − 8 Incomplete- 3	17.5 ± 13.3	Electrical stimulation	Biphasic pulses, frequency 30 Hz, pulse duration 300 µs, amplitude 30–100 mA.	Rectus abdominis, obliquus externus abdominis and erector spinae muscles.	Electromyography, trunk stability measured through reaching tasks, Isokinetic test on dynamometer.	Induced trunk muscle activation, trunk stability increased with increased reaching distance.
(Gorgey and Gouda 2022)	Case report	n = 1; M	25	T3; AIS - A	3.8	Epidural spinal electrical stimulation	Frequency 20 Hz, pulse width 240 µs, amplitude of the current gradually increased from 0–10 V.	T11–T12 vertebral region.	Electromyography, perturbation of trunk control.	Activation of abdominal muscles, immediate restoration of trunk control during seated position.
(Rowald et al. 2022)	Clinical trial	n = 3; M	34.0 ± 6.2	T4 - T7; AIS A − 1 AIS B − 2	4.3 ± 4.1	Epidural spinal electrical stimulation	Frequency 70–80 Hz, single pulses (0.5 Hz) were delivered at increasing amplitude to elicit muscle responses.	L1 and L2 spinal segments.	Inspecting muscular activity and kinematics, activity-specific stimulation programs, quantification of muscle mass.	Motor neurons innervating the trunk and abdominal musculatures were activated and facilitated improved trunk posture.
(Friederich et al. 2022)	Feasibility study	n = 5; M = 3 F = 2	46.8 ± 9.0	C5 - T10; AIS A − 3 AIS B − 1 AIS C − 1	13.2 ± 6.6	Functional neuromuscular stimulation	Frequency 40 Hz, pulse 250 µs and amplitude set at 20mA.	Applied to nerves innervating the erector spinae, quadratus lumborum, adductor magnus, gluteus maximus, gluteus medius, and hamstring semimembranosus.	Trunk angles measured using motion capture system, postural sway, reaching movements.	Postural sway reduced, reaching ability increased, time required for maintaining upright posture improved.

### Participants characteristics

**Fig. 4:**
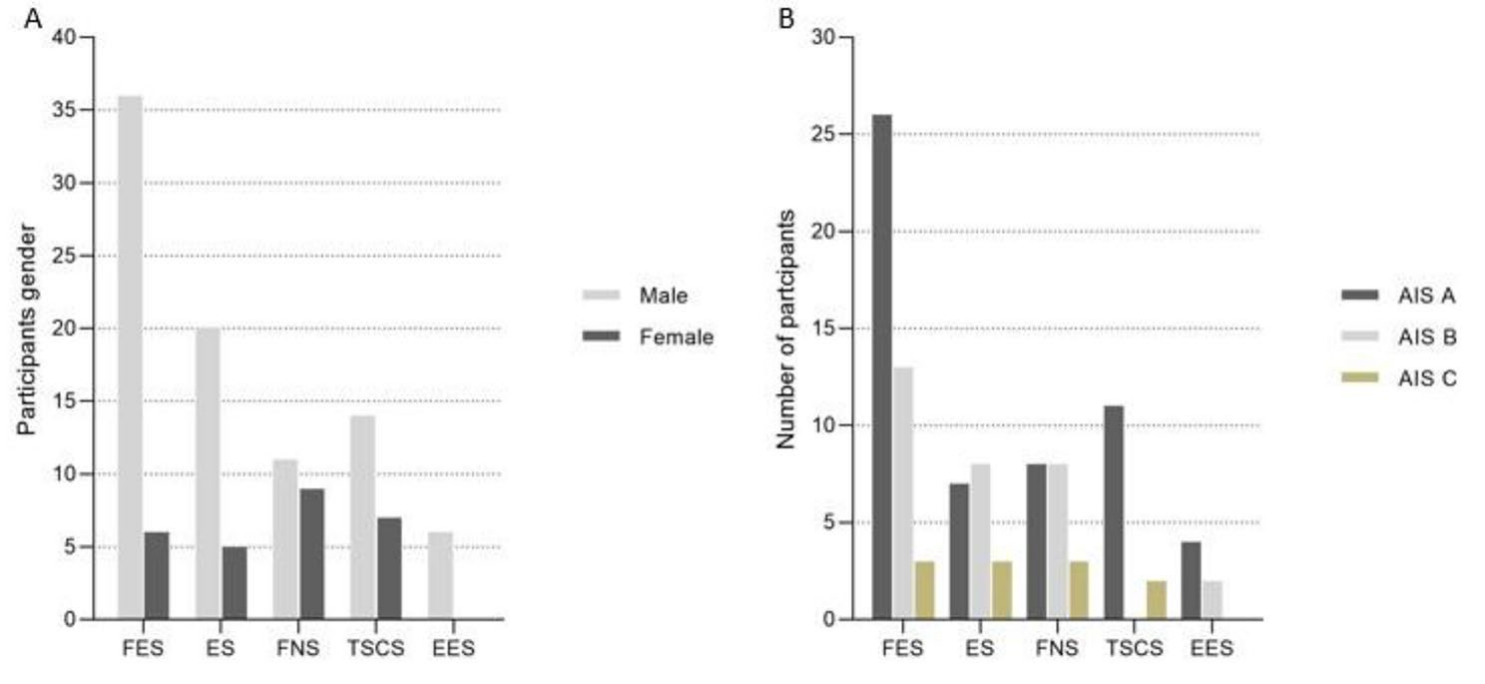
For specific neuromodulation techniques: (**A**) participants gender classification; and (**B**) participants AIS scores. Abbreviation: FES = functional electrical stimulation; ES = electrical stimulation; FNS = functional neuromuscular stimulation; TSCS = transcutaneous electrical spinal cord stimulation; EES = epidural spinal electrical stimulation; AIS = American Spinal Injury Association Impairment Scale

Table 1 shows the participants’ characteristics. Majority of the articles (9 of 21 articles) included 4–6 individuals with SCI to investigate the effects of a given neuromodulation technique. Only three included articles presented results from 10 or more participants. Overall, there were 87 male and 27 female participants. One included article did not report the gender of three participants. Of these 117 individuals (Fig. 4A), 36 men and 6 women participated in studies investigating FES. A total of 20 men and 5 women involved ES studies. Similarly, FNS studies involved 11 male and 9 female participants, TSCS studies recruited 14 men and 7 women, whereas EES studies involved 6 male participants. Of 21 included articles, 14 articles recruited both men and women, although most of them involved a larger proportion of male participants. Six included articles only involved male participants, whereas one article involved only a female. Given the smaller number of participants, it is difficult to draw conclusions on the effectiveness of these neuromodulation techniques. However, this review provides an overview on the potential effects of these treatments.

Approximately half of the included studies (10 out of 21 articles) had participants with SCI with the injury level between cervical and thoracic regions. However, the severity and neurological level of SCI varied from one person to another. Six included studies recruited participants only with cervical SCI, while one included article did not report the participants’ injury level. Nineteen included articles described the AIS grades, where the participants were classified as AIS A = 56; AIS B = 31; and AIS C = 11. Two included articles did not specify the AIS grade. In addition, Fig. 4B reveals that FES studies had the highest number of participants (42 individuals; AIS A = 26; AIS B = 13; and AIS C = 3), followed by FNS studies (19 participants; AIS A = 8; AIS B = 8; and AIS C = 3). EES studies had the lowest number of participants with SCI, with 4 AIS A; and 2 AIS B.

### Stimulation characteristics and protocol

Of the all included studies, nine articles used a stimulation device which was implanted at a specific location targeting the spinal nerves, whereas three articles used electrodes that were placed on the spinal cord over the targeted neural network. T12-L1 or L1-L2 spinal levels were the most common sites to implant the stimulation device or for spinal electrode placements. These sites were chosen to stimulate the nerves innervating the trunk and leg muscles. The stimulation protocol of the implanted stimulation device included biphasic stimulation with pulse durations ranging from 0 to 300 µs and frequencies ranging from 20 to 30 Hz, with the intensity being gradually increased to elicit muscle responses. The transcutaneous spinal stimulation method used monophasic or biphasic rectangular pulses with frequencies between 15 and 30 Hz, intensities ranging from 90 to 150 mA, and a carrier frequency of 10 kHz. The remaining nine included articles directly placed surface electrodes over the targeted muscles for stimulation. Muscles of the trunk, abdomen, back, hip, and legs were specifically stimulated through this transcutaneous muscle stimulation technique using biphasic stimulation, with frequencies between 30 and 35 Hz, pulse width ranging from 300 to 400 µs, and intensities that caused strong visible muscle contractions.

### Primary measures

Table 1 demonstrates that eight included studies used functional reaching tasks, sitting stability, and sitting posture as their primary outcome measures, whereas 11 included studies primarily evaluated wheelchair propulsion ability, trunk range of motion, the extent of trunk tilting, and trunk stability. Additionally, two included studies used functional trunk assessments and sitting function evaluations to assess the improvement in trunk and sitting functions in individuals with SCI. Approximately half of included articles (11 out of 21 articles) investigated muscle activation, muscle tone, and muscle contraction as measured by surface electromyography (EMG). The increased EMG activities of trunk and back muscles after neuromodulation was considered as a sign of improvement. In addition, 7 out of 21 included articles evaluated the post-stimulation changes in sitting stability, sitting balance, trunk stability, trunk tilt, or trunk perturbations using a motion capture system and/or force plate data. Moreover, two articles assessed the ability of the trunk in performing activities of daily living (such as feeding, bathing, dressing, grooming, etc.), indoor and outdoor mobility tasks (such as transferring and rolling in bed), and using the toilet.

### Treatment effects

For the five included FES studies, two showed improved sitting posture with increased reaching distance, and improved ability to perform transfers. Three FES studies found increased trunk and abdominal muscle tone and elevated muscle contraction, which suggest improved trunk stability. Four studies revealed improved forward reach distance with more erect sitting posture following ES, while one study demonstrated an increase in EMG amplitude of trunk muscles with improved trunk stability after ES. For the five FNS studies, two found improved trunk tilt and increased trunk range of motion with an improved erect sitting posture. Another two studies showed increased activation of paraspinal and hip muscles, while the remaining study revealed reduced sitting postural sway with increased duration of maintaining an upright sitting posture. For the three TSCS articles, two studies exhibited post-treatment elevated trunk muscle activity and improved trunk and sitting posture, while one study reported improved post-TSCS static and dynamic sitting balance. For the three EES studies, two studies indicated an activated muscular response and facilitated trunk movements in a sitting position after EES. The remaining study found increased reaching distance and improved reaching performance following EES.

### Adverse effects

The adverse effects resulting from a specific type of neuromodulation were not well defined in the majority of the included studies. However, a few studies reported some common adverse effects, such as muscle fatigue after FES or ES treatments (Triolo et al. 2013a; Kouwijzer et al. 2022), pain and autonomic dysreflexia developed following TSCS (Rath et al. 2018), and a sudden increase in blood pressure, and skin rashes during the TSCS intervention (Tharu et al. 2022b).

## Discussion

This review provides a comprehensive overview on the existing literature regarding neuromodulation (FES, ES, FNS, TSCS, and EES) in order to better guide future studies. Although individuals with SCI consider trunk recovery as a great priority in terms of quality of life (Looft et al. 2022), it has received insufficient attention in rehabilitation or research. In the last two decades, research has shown that paralyzed individuals may regain considerable amount of voluntary control in standing, walking, leg motions (Rath et al. 2018; Alam et al. 2020), and trunk movement (Tharu et al. 2022b) by stimulating the spinal neuronal circuitries caudal to the site of damage. The findings of this review provide preliminary support for the notion that stimulation may increase muscle tone and activation, which may lead to enhanced motor or functional performance (Rath et al. 2018).

This review identified 21 articles demonstrating the potential of various neuromodulation techniques in improving trunk and sitting functions following SCI. It was found that the majority of the included studies were case series or case study. Since they provided preliminary evidence for the post-neuromodulation improvements in trunk and sitting improvements through sensorimotor recovery, future studies should determine the effects of specific neuromodulation technique on trunk control and activities of daily living such as dressing, wheelchair propulsion, hygiene, and self-care (Triolo et al. 2009). In addition, the results of this review indicated that large-scale randomized controlled trials are warranted to compare the effectiveness of various types of neuromodulation techniques on trunk control, sitting balance, and functional performance of people with SCI.

People with SCI have a strong desire to regain the ability to move their trunk, while keeping a stable posture (Bao et al. 2020). Trunk muscles are essential for maintaining the required trunk stability while carrying out daily activities. The trunk has to move in various directions to facilitate both activities of daily living and functional motions. Thus, the ability to reach forward without considering other factors may not be a reliable indicator of a person’s functional capacity (Gao et al. 2015). This may limit our understanding of the functional recovery of trunk and sitting functions after SCI (Keller et al. 2021). The improvements in functional task performance in sitting have been linked to increased trunk control, making this a promising early predictor of success in rehabilitation (Field-Fote and Ray 2010). However, these muscles were rarely the major focus of rehabilitation regimens and were rarely included in assessments that are often used to categorize motor function in people with SCI (Momeni et al. 2016). Findings from one of our included studies supported that improved trunk and sitting mobility skills after the TSCS intervention (Tharu et al. 2022b). The findings highlight the importance of assessments and training of trunk muscles in rehabilitation programs.

Our review suggests that five forms of neuromodulation techniques were feasible for assisting people with SCI to improve their trunk and sitting stability. The promising results support that neuromodulation could be used as an alternative to traditional therapy for sensorimotor recovery (Lin et al. 2022), lower and upper extremity functional restoration (Alam et al. 2020; McGeady et al. 2022), and improvement of trunk and sitting functions in people with SCI (Tharu et al. 2022b). However, trunk function recovery after SCI may vary greatly among individuals depending on the level, severity, and duration following injury (Milosevic et al. 2015b). In addition to stimulation parameters and settings, different neuromodulation methods (FES, ES, FNS, TSCS, and EES) may be influenced by the above-mentioned factors to different extents. Therefore, future studies should report clinical outcomes based on the characteristics of injury, SCI classification, and neurostimulation parameters so as to assist the clinicians and researchers in establishing standard stimulation parameters for various conditions. Although prior small-scale case studies have revealed the possibility of using electrical stimulation to stabilize the trunk for restoring the erect sitting posture (Wilkenfeld et al. 2006a; Lambrecht et al. 2009), there is no conclusion regarding its effectiveness. Future research should focus on the efficacy of specific neuromodulation methods in trunk control recovery.

FES, TSCS, and EES studies usually reported improved trunk functions, such as reaching tasks, sitting balance, perturbation, trunk tilt, which are closely related to activities of daily living. Conversely, ES and FNS studies mainly assessed sitting posture, or quantifying muscle mass and its properties which were less functional. Therefore, FES, TSCS, and EES may be the preferred neuromodulation therapies because they improve functions in people with SCI. Of these three neuromodulation methods, FES was mainly used to improve specific muscle function (Rahimi et al. 2020), while TSCS and EES were used for sensorimotor improvement of the targeted neural circuit (Rath et al. 2018; Gorgey and Gouda 2022). FES was mainly investigated in individuals with incomplete SCI, where they had some preserved motor functions (Armstrong et al. 2018; Bergmann et al. 2019), whereas TSCS and EES have been found to be effective in both complete and incomplete SCI (Gill et al. 2020; Tharu et al. 2022b). It was also reported that TSCS could produce the same effects as EES (Rahman et al. 2022). As such, TSCS appears to be a more popular neuromodulation treatment given its non-invasive procedures (Rahman et al. 2022). Collectively, the choice of specific neuromodulation therapies depends on the clinical symptoms and rehabilitation requirements of the affected individual. Specifically, FES could be beneficial for people with AIS (grades B - D) where the goal is to increase muscle power. However, TSCS could be used to improve sensory and motor functions for people with all categories of SCI.

Although the exact mechanism for the reported trunk and sitting control recovery after neuromodulation in people with SCI has not been fully understood, different neuromodulation methods have slightly different suggested mechanisms for the observed improvements. FES primarily uses tonic co-contraction of the trunk muscles in an attempt to promote trunk stiffness and enhance sitting posture (Patel et al. 2017). ES may increase the excitability of the dormant circuitry, increasing the possibility of motor activity in response to supraspinal drive supplied through sparing descending axons (Keller et al. 2021). FNS may contribute to the trunk’s stiffening by raising intra-abdominal pressure, which has been linked to improvements in spinal stability, similar to the concept of wearing an abdominal belt to increase lumbar stiffness (Friederich et al. 2022). TSCS may enable spinal postural-specific neural networks that could reliably act as feed-forward mechanism to keep the body in balance at both sub- and possibly supralesional levels (Rath et al. 2018). Furthermore, EES recruits large-diameter afferent fibers when it enters the spinal cord through dorsal roots. The recruited fibers activate the motor neurons supplied by the dorsal roots located in the spinal segment. Therefore, targeting individual dorsal roots may modulate specific motor neurons (Rowald et al. 2022).

Individuals with SCI are at risk of falls given their trunk instability. Rehabilitation programs should focus on improving sitting balance to lower the risk of fall incidents. As such, being able to improve one’s trunk control may help lessen such risk ((Tharu et al. 2022b). A study showed that stimulating the trunk and hip extensor muscles by implanted ES could improve the sitting posture and enabled forward reaching ((Triolo et al. 2013b). Likewise, a recent study reported improved trunk and sitting ability following TSCS ((Tharu et al. 2022b). These findings suggest that ES and TSCS may be used as adjunct treatment to prevent falls in people with SCI if randomized controlled trial can prove the long-term efficacy of these neuromodulation methods in improving trunk function and minimize falls.

This review study was limited to original relevant research published in English. Although this review showed promising results for the use of neuromodulation therapy to target trunk control and sitting recovery after SCI, evidence in this area is still accumulating. The stimulation parameters, such as electrode positions, frequency, and amplitude, are not standardized. The stimulation parameters may vary depending on the desired outcomes. It was also difficult to compare the outcomes of different articles because of the diverse intervention dosages and stimulation method. It was noted that there was insufficient information regarding the stimulation duration and follow-up protocols in previous studies. Future studies in this field should provide more details about the stimulations protocols and post-treatment follow-up protocols. To be used in clinics, further research is warranted to standardize the neuromodulation of the trunk and rehabilitation for people with SCI.

## Conclusions

This review provides preliminary evidence to support the effects of neuromodulation therapies on restoring trunk control and sitting abilities in people with SCI. Among five types of neuromodulation methods, FES and TSCS techniques could be more suitable in clinical practice for people with incomplete and complete SCI, respectively. Multiple factors should be considered to determine the efficacy of specific neuromodulation therapies in improving the recovery of trunk and sitting functions in these people. Future pilot randomized controlled trials should be conducted to evaluate the feasibility of evaluating the effects of various stimulation parameters on trunk control, sitting balance, and adverse effects. If these trails are found to be feasible, fully powered randomized controlled trials are warranted to facilitate the translation of research findings into clinical practice to benefit people with SCI.

## Data Availability

Not applicable.

## Abbreviations

SCI: Spinal cord injury
FES: Functional electrical stimulation
ES: Electrical stimulation
FNS: Functional neuromuscular stimulation
TSCS: Transcutaneous electrical spinal cord stimulation
EES: Epidural spinal electrical stimulation
AIS: American Spinal Injury Association Impairment Scale
RCT: Randomised controlled trial
EMG: Electromyography

## References

[CR1] Alam M, Ling YT, Wong AY, Zhong H, Edgerton VR, Zheng YP (2020). Reversing 21 years of chronic paralysis via non-invasive spinal cord neuromodulation: a case study. Ann Clin Transl Neurol.

[CR2] Armstrong KL, Lombardo LM, Foglyano KM, Audu ML, Triolo RJ (2018). Automatic application of neural stimulation during wheelchair propulsion after SCI enhances recovery of upright sitting from destabilizing events. J Neuroeng Rehabil.

[CR3] Audu ML, Lombardo LM, Schnellenberger JR, Foglyano KM, Miller ME, Triolo RJ (2015). A neuroprosthesis for control of seated balance after spinal cord injury. J Neuroeng Rehabil.

[CR4] Bao X, Friederich AR, Audu ML, Triolo RJ. (2020). “An integrated control system for optimal human trunk motion”, in: 2020 8th IEEE RAS/EMBS International Conference for Biomedical Robotics and Biomechatronics (BioRob): IEEE). 1055–1060. 10.1109/BioRob49111.2020.9224363.

[CR6] Bergmann M, Zahharova A, Reinvee M, Asser T, Gapeyeva H, Vahtrik D (2019). The effect of functional electrical stimulation and therapeutic exercises on trunk muscle tone and dynamic sitting balance in persons with chronic spinal cord injury: a crossover trial. Medicina.

[CR5] Bergmann M, Zahharova A, Ereline J, Asser T, Gapeyeva H, Vahtrik D (2020). Single session exercises and concurrent functional electrical stimulation are more effective on muscles’ force generation than only exercises in spinal cord injured persons: a feasibility study. J Musculoskel Neuronal Interact.

[CR7] Chiou S-Y, Strutton PH (2020). Crossed corticospinal facilitation between arm and trunk muscles correlates with trunk control after spinal cord injury. Front Hum Neurosci.

[CR8] Field-Fote EC, Ray SS (2010). Seated reach distance and trunk excursion accurately reflect dynamic postural control in individuals with motor-incomplete spinal cord injury. Spinal Cord.

[CR9] Friederich AR, Audu ML, Triolo RJ (2020). Characterization of the force production capabilities of paralyzed trunk muscles activated with functional neuromuscular stimulation in individuals with spinal cord injury. IEEE Trans Biomed Eng.

[CR10] Friederich AR, Bao X, Triolo RJ, Audu ML. Feedback control of upright seating with functional neuromuscular stimulation during a functional task after spinal cord injury: a case study. 2021 43rd Annual International Conference of the IEEE Engineering in Medicine & Biology Society (EMBC): IEEE. 2021;5719–22. 10.1109/EMBC46164.2021.9629582. 10.1109/EMBC46164.2021.9629582 34892419

[CR11] Friederich AR, Bao X, Triolo RJ, Audu ML (2022). Feedback control of upright seating with functional neuromuscular stimulation during a reaching task after spinal cord injury: a feasibility study. J Neuroeng Rehabil.

[CR12] Gao KL, Chan K, Purves S, Tsang WW (2015). Reliability of dynamic sitting balance tests and their correlations with functional mobility for wheelchair users with chronic spinal cord injury. J Orthop Translation.

[CR13] Gill M, Linde M, Fautsch K, Hale R, Lopez C, Veith D (2020). Epidural electrical stimulation of the lumbosacral spinal cord improves trunk stability during seated reaching in two humans with severe thoracic spinal cord injury. Front Syst Neurosci.

[CR14] Gorgey AS, Gouda JJ (2022). Single lead epidural spinal cord stimulation targeted trunk control and standing in complete paraplegia. J Clin Med.

[CR15] Greiner N, Barra B, Schiavone G, Lorach H, James N, Conti S (2021). Recruitment of upper-limb motoneurons with epidural electrical stimulation of the cervical spinal cord. Nat Commun.

[CR16] Johnston TE. NMES and FES in Patients with Neurological Diagnoses. Modalities for Therapeutic Intervention. In: Bellew JW, Michlovitz SL, Nolan Jr. TP.eds, 6e, McGraw Hill, 2016.

[CR17] Kapadia NM, Zivanovic V, Furlan J, Craven BC, McGillivray C, Popovic MR (2011). Functional electrical stimulation therapy for grasping in traumatic incomplete spinal cord injury: randomized control trial. Artif Organs.

[CR18] Keller A, Singh G, Sommerfeld JH, King M, Parikh P, Ugiliweneza B (2021). Noninvasive spinal stimulation safely enables upright posture in children with spinal cord injury. Nat Commun.

[CR19] Kouwijzer I, van der Meer M, Janssen TW (2022). Effects of trunk muscle activation on trunk stability, arm power, blood pressure and performance in wheelchair rugby players with a spinal cord injury. J Spinal Cord Med.

[CR20] Kukke SN, Triolo RJ (2004). The effects of trunk stimulation on bimanual seated workspace. IEEE Trans Neural Syst Rehabil Eng.

[CR21] Lambrecht JM, Audu ML, Triolo RJ, Kirsch RF (2009). Musculoskeletal model of trunk and hips for development of seated-posture-control neuroprosthesis. J Rehabil Res Dev.

[CR22] Laskin JJ, Waheed Z, Thorogood NP, Nightingale TE, Noonan VK (2022). Spinal cord stimulation research in the restoration of motor, sensory and autonomic function for individuals living with spinal cord injuries: a scoping review. Arch Phys Med Rehabil.

[CR23] Lin A, Shaaya E, Calvert JS, Parker SR, Borton DA, Fridley JS (2022). A review of functional restoration from spinal cord stimulation in patients with spinal cord injury. Neurospine.

[CR24] Looft JM, Sjoholm R, Hansen AH, Fairhurst S, Voss G, Dellamano CA (2022). User-centered design and development of a trunk control device for persons with spinal cord injury: a pilot study. J Spinal Cord Med.

[CR25] McGeady C, Vučković A, Tharu NS, Zheng Y-P, Alam M (2022). Brain-computer interface priming for cervical transcutaneous spinal cord stimulation therapy: an exploratory case study. Front Rehabilitation Sci.

[CR26] Milosevic M, Masani K, Kuipers MJ, Rahouni H, Verrier MC, McConville KM (2015). Trunk control impairment is responsible for postural instability during quiet sitting in individuals with cervical spinal cord injury. Clin Biomech Elsevier Ltd.

[CR27] Milosevic M, Masani K, Wu N, McConville KM, Popovic MR (2015). Trunk muscle co-activation using functional electrical stimulation modifies center of pressure fluctuations during quiet sitting by increasing trunk stiffness. J Neuroeng Rehabil.

[CR28] Momeni K, Canton S, Ramanujam A, Garbarini E, Forrest GF. (2016). “Effects of lower limb electrical stimulation on trunk stability in persons with SCI during walking: a case series”, in: 2016 38th Annual International Conference of the IEEE Engineering in Medicine and Biology Society (EMBC): IEEE). 6377–6380. 10.1109/EMBC.2016.7592187. 10.1109/EMBC.2016.7592187 28269707

[CR29] Patel K, Milosevic M, Nakazawa K, Popovic MR, Masani K (2017). Wheelchair neuroprosthesis for improving dynamic trunk stability. IEEE Trans Neural Syst Rehabil Eng.

[CR30] Rahimi M, Torkaman G, Ghabaee M, Ghasem-Zadeh A (2020). Advanced weight-bearing mat exercises combined with functional electrical stimulation to improve the ability of wheelchair-dependent people with spinal cord injury to transfer and attain independence in activities of daily living: a randomized controlled trial. Spinal Cord.

[CR31] Rahman MA, Tharu NS, Gustin SM, Zheng Y-P, Alam M (2022). Trans-spinal electrical stimulation therapy for functional rehabilitation after spinal cord injury. J Clin Med.

[CR32] Rath M, Vette AH, Ramasubramaniam S, Li K, Burdick J, Edgerton VR (2018). Trunk stability enabled by noninvasive spinal electrical stimulation after spinal cord injury. J Neurotrauma.

[CR33] Rowald A, Komi S, Demesmaeker R, Baaklini E, Hernandez-Charpak SD, Paoles E (2022). Activity-dependent spinal cord neuromodulation rapidly restores trunk and leg motor functions after complete paralysis. Nat Med.

[CR34] Seáñez I (2021). Motor improvements enabled by spinal cord stimulation combined with physical training after spinal cord injury: review of experimental evidence in animals and humans. Bioelectronic Med.

[CR35] Tharu NS, Alam M, Bajracharya S, Chaudhary GP, Pandey J, Kabir MA. (2022a). Caregivers’ knowledge, attitude, and practice towards pressure injuries in spinal cord injury at rehabilitation center in Bangladesh. Advances in Orthopedics. 2022. 10.1155/2022/8642900. 10.1155/2022/8642900 PMC9213162 35747167

[CR36] Tharu NS, Alam M, Ling YT, Wong AY, Zheng Y-P (2022). Combined Transcutaneous Electrical spinal cord stimulation and Task-Specific Rehabilitation improves trunk and sitting functions in people with chronic Tetraplegia. Biomedicines.

[CR40] Triolo RJ, Boggs L, Miller ME, Nemunaitis G, Nagy J, Bailey SN (2009). Implanted electrical stimulation of the trunk for seated postural stability and function after cervical spinal cord injury: a single case study. Arch Phys Med Rehabil.

[CR37] Triolo RJ, Bailey SN, Lombardo LM, Miller ME, Foglyano K, Audu ML (2013). Effects of intramuscular trunk stimulation on manual wheelchair propulsion mechanics in 6 subjects with spinal cord injury. Arch Phys Med Rehabil.

[CR39] Triolo RJ, Bailey SN, Miller ME, Lombardo LM, Audu ML (2013). Effects of stimulating hip and trunk muscles on seated stability, posture, and reach after spinal cord injury. Arch Phys Med Rehabil.

[CR42] Wilkenfeld A, Audu M, Triolo R (2006). Feasibility of a neuroprosthesis for the control of seated posture after spinal cord injury with functional electrical stimulation: a simulation study. J Rehabilitation Res Dev.

[CR43] Wilkenfeld AJ, Audu ML, Triolo RJ. Feasibility of functional electrical stimulation for control of seated posture after spinal cord injury: a simulation study. J Rehabilitation Res Dev. 2006b;43(2). 10.1682/JRRD.2005.06.0101. 10.1682/jrrd.2005.06.0101 16847781

[CR44] Yang Y-S. Effects of functional electrical stimulation on trunk musculature during wheelchair propulsion. University of Pittsburgh; 2005. 10.1177/1545968308331145 19261768

[CR38] 10.1016/j.apmr.2013.04.010.

[CR41] 10.1016/j.apmr.2008.07.029.

