# Correction: Neuromodulation for recovery of trunk and sitting functions following spinal cord injury: a comprehensive review of the literature

**DOI:** 10.1186/s42234-023-00116-3

**Published:** 2023-06-29

**Authors:** Niraj Singh Tharu, Arnold Yu Lok Wong, Yong-Ping Zheng

**Affiliations:** 1 Department of Biomedical Engineering, The Hong Kong Polytechnic University, Hong Kong SAR, China; 2 Department of Rehabilitation Sciences, The Hong Kong Polytechnic University, Hong Kong SAR, China; 3 Research Institute for Smart Ageing, TheHong Kong Polytechnic University, Hong Kong SAR, China

**Correction: Bioelectron Med 9, 11 (2023)**

**https://doi.org/10.1186/s42234-023-00113-6**

Following publication of the original article (Tharu et al. 2023), the authors identified errors in Fig. 3 and Fig. 4. The correct figures are given below.

**Fig. 3:**
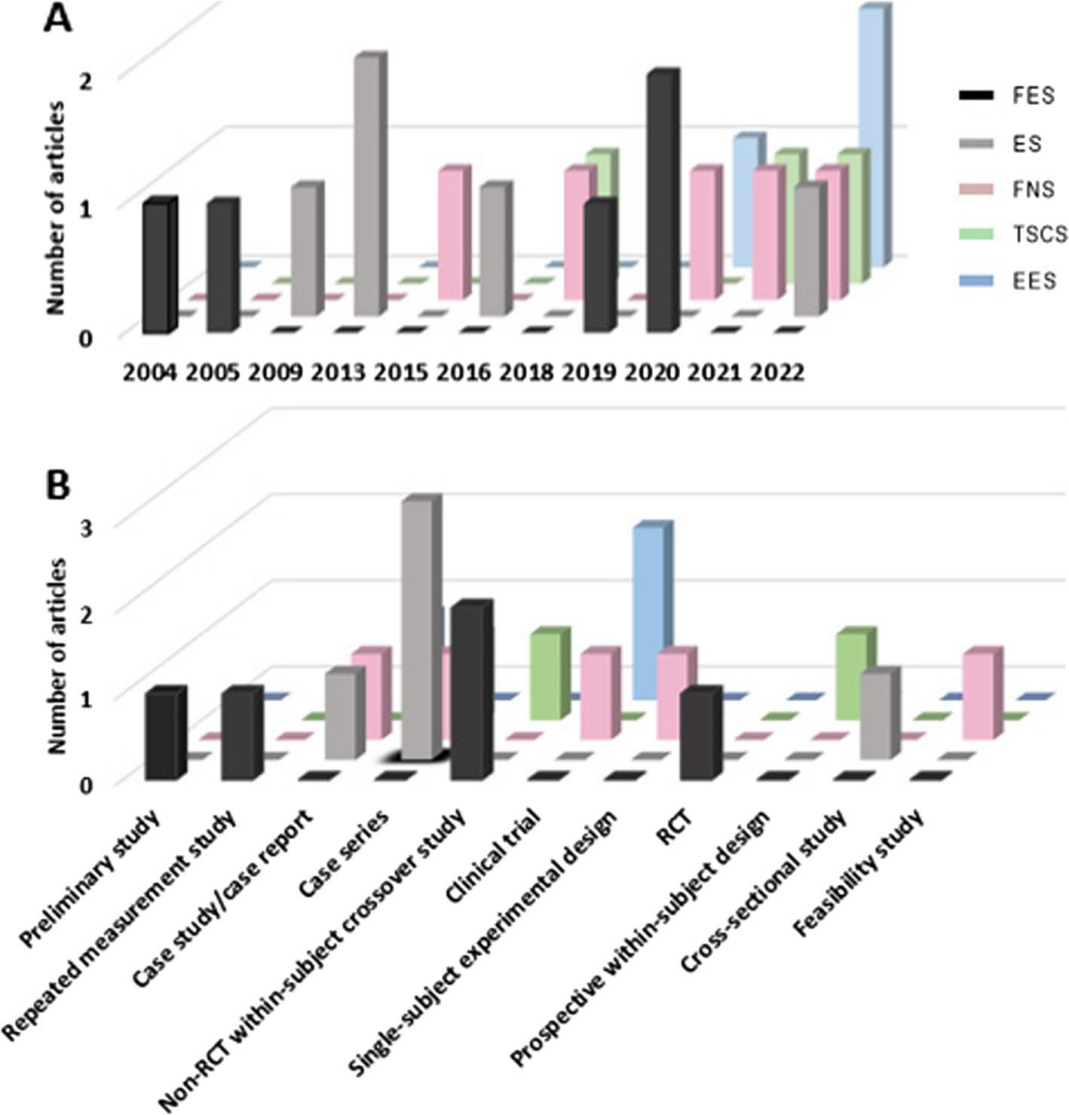
**A** Publication trends for FES (*n* = 5), ES (*n* = 5), FNS (*n* = 5), TSCS (*n* = 3), and EES (*n* = 3) by year; and **B** reported study designs of each neuromodulation technique with number of articles published. Abbreviation: FES = functional electrical stimulation; ES = electrical stimulation; FNS = functional neuromuscular stimulation; TSCS = transcutaneous electrical spinal cord stimulation; EES = epidural spinal electrical stimulation; RCT = randomized controlled trial

**Fig. 4:**
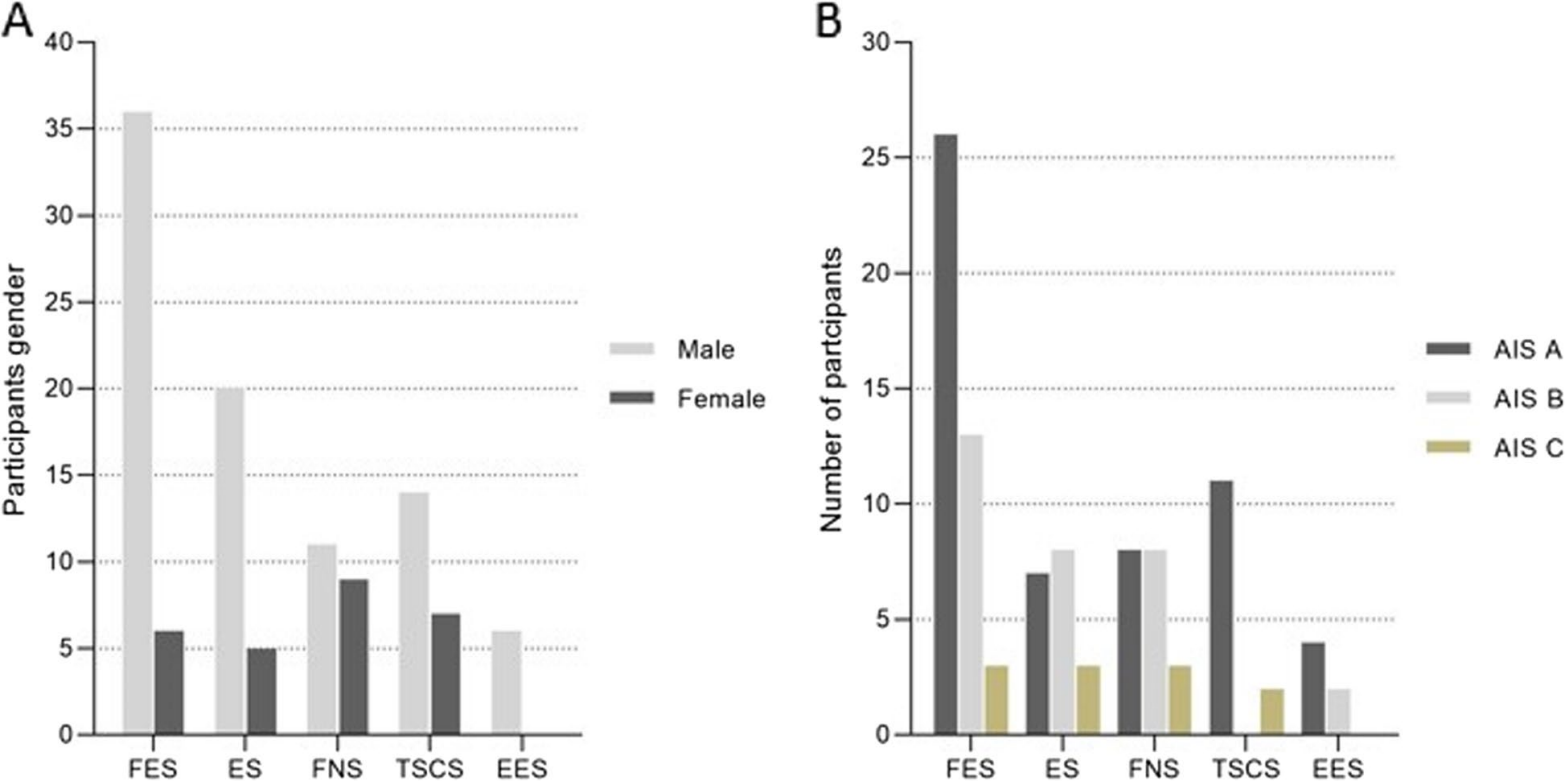
For specific neuromodulation techniques: **A** participants gender classification; and **B** participants AIS scores. Abbreviation: FES = functional electrical stimulation; ES = electrical stimulation; FNS = functional neuromuscular stimulation; TSCS = transcutaneous electrical spinal cord stimulation; EES = epidural spinal electrical stimulation; AIS = American Spinal Injury Association Impairment Scale

The original article (Tharu et al. 2023) has been corrected.
